# Efficacy, Safety, and Outcomes following Accelerated and Iontophoresis Corneal Crosslinking in Progressive Keratoconus

**DOI:** 10.3390/jcm12082931

**Published:** 2023-04-18

**Authors:** Sami Saad, Rana Saad, Isabelle Goemaere, Roxane Cuyaubere, Marie Borderie, Vincent Borderie, Nacim Bouheraoua

**Affiliations:** 1 CHNO Des Quinze-Vingts, IHU ForeSight, INSERM-DGOS CIC 1423, 28 Rue de Charenton, F-75012 Paris, France; 2 Sorbonne Université, INSERM, CNRS, Institut de la Vision, 17 rue Moreau, F-75012 Paris, France

**Keywords:** cornea, keratoconus, corneal collagen crosslinking, iontophoresis crosslinking, accelerated crosslinking

## Abstract

Purpose: To investigate the outcomes of accelerated (A-CXL) and iontophoresis (I-CXL) corneal crosslinking in a large retrospective cohort with progressive keratoconus. Methods: This retrospective observational cohort study included consecutive patients treated by A-CXL (9 mW/5.4 J/cm^2^) or I-CXL with a minimal follow-up of 12 months. Visual acuity, manifest refraction, topography, specular microscopy, and corneal optical coherence tomography (OCT) were evaluated at baseline and at the last visit. Progression was defined as an increase in the maximum topographic keratometry (Kmax) of 1D. Results: 302 eyes of 241 patients with a mean age of 25.2 ± 7.5 years were included from 2012 to 2019: 231 and 71 eyes in the A-CXL and I-CXL groups, respectively. The mean follow-up was 27.2 ± 13.2 months (maximum: 85.7 months). Preoperatively, the mean Kmax was 51.8 ± 4.0D, with no differences between groups. Mean topographic measurements and spherical equivalent remained stable during the follow-up. At the last visit, CXL failure was reported in 60 eyes (19.9%): 40 (14.7%) versus 20 (28.2%) in A-CXL versus I-CXL, respectively, *p* = 0.005. The likelihood of progression after CXL was significantly higher following I-CXL: RR = 1.62, CI95 = [1.02 to 2.59], *p* = 0.04. Demarcation line presence at 1 month was positively correlated with higher efficacy of CXL, *p* = 0.03. No endothelial damage was reported, especially in 51 thin corneas (range = 342–399 µm). Conclusions: A-CXL seems more effective than I-CXL in stabilizing keratoconus; this is to be taken into account when a therapeutic indication is posed according to the aggressiveness of the keratoconus.

## 1. Introduction

Keratoconus (KC) is a noninflammatory progressive degenerative corneal disorder characterized by progressive corneal thinning, protrusion, and visual impairment [1]. Corneal crosslinking (CXL) was introduced in 2003 by Wollensak and colleagues [2], aiming at slowing or halting KC progression by strengthening the cornea through induction of chemical bonds or “cross-links” via a photochemical reaction using riboflavin and ultraviolet-A (UVA) light. The gold standard CXL protocol (S-CXL or Dresden protocol) is a time-consuming epithelium-off procedure. Numerous alternative protocols have been suggested in order to reduce operative time, such as accelerated CXL (A-CXL), where a higher irradiance of UVA is delivered over a reduced time [3], or iontophoresis transepithelial CXL (I-CXL) [4], where the penetration of ionized riboflavin through the intact epithelium is improved by the application of an electric current, decreasing the likelihood of complications related to epithelial debridement. I-CXL protocol efficacy remains unclear to date. Jouve et al. found that I-CXL is less effective than epi-off S-CXL in 80 eyes over 24 months, with a 20% failure rate after I-CXL and 7.5% after S-CXL. [5]. However, Bikbova et al. showed that I-CXL is a viable alternative in a 24-month follow-up randomized control trial including 149 eyes [6]. In a recent 7-year prospective study, Vinciguerra et al. reported a 26% progression rate after I-CXL [7]. Whereas, in a large 5-year prospective study on A-CXL, 8% of the eyes had a Kmax progression of 1 D within the 2nd–3rd year follow-up visits [8].

The aim of this large retrospective cohort study was to describe outcomes following A-CXL and I-CXL protocols for progressive keratoconus.

## 2. Methods

This non-randomized retrospective study with consecutive recruitment included patients who were treated with A-CXL or I-CXL for progressive KC in the Quinze-Vingts National Ophthalmology Hospital (Paris, France) from 1 January 2012, to 31 March 2019. The inclusion criteria were patients with progressive keratoconus defined as a maximal keratometry progression (Kmax) superior to 1.0 D and a clear cornea. The exclusion criteria were a follow-up period <12 months, ocular or systemic disease that may affect the cornea, intracorneal ring segment implantation (ICRS), and pregnancy or breastfeeding. CXL failure was defined as an increase in Kmax of 1.00 D or more in less than 12 months. Indication of A-CXL or I-CXL was assessed in all cases by a cornea specialist. The choice of the CXL protocol was based on the clinician’s appreciation. The tenets of the Declaration of Helsinki were respected, and this study was approved by the Ethics Committee of the French Society of Ophthalmology (Institutional Review Board 00008855). Informed consent was obtained from patients before surgery.

### 2.1. Surgical Procedure

#### 2.1.1. Accelerated Corneal Collagen CXL

After epithelial debridement, 0.1% riboflavin solution (Ricrolin, Sooft SPA, Montegiorgio, Italy) is applied for 15 min. Next, the cornea is irradiated with a 9 mW/cm² UV-A light (X-Vega, Sooft, SPA) for 10 min at a 5 cm distance.

#### 2.1.2. Iontophoresis Corneal Collagen CXL

A 1.0 mA electric current is applied for 5 min using the iontophoresis device (I-ON CXLr, Iacer, Veneto, Italy) to allow the penetration of riboflavin (Ricrolin+, Sooft SPA). Then, the cornea is irradiated with a 9 mW/cm² UV-A light (X-Vega, Sooft, SPA) for 10 min at a 5 cm distance.

#### 2.1.3. Data Collection

At the preoperative visit and last visit, the following data were collected: refraction, best-corrected visual acuity (BCVA) with glasses, corneal topography using Scheimpflug imaging (Pentacam, Oculus Inc., Wetzlar, Germany), corneal pachymetry, and anterior segment optical coherence tomography (AS-OCT) (Optovue Inc, Fremont, CA, USA) outcomes such as the presence of a postoperative demarcation line (DL), endothelial cell density (ECD) (cells/mm²) measured by no-contact specular microscopy, and any adverse event linked to the CXL procedure.

#### 2.1.4. Statistical Analysis

Descriptive statistics were reported as mean ± standard deviation (SD) for continuous variables and as a percentage for categorical variables. We used the d’Agostino–Pearson test to assess the normal distribution of our data. For binary outcomes, the chi-square test was used for intergroup comparisons of proportions. Student’s *t*-test for paired data was used to compare preoperative and postoperative continuous parameters. Measurements between groups were compared using an independent sample *t*-test. The Snellen BCVA was converted to a logarithm of the minimum angle of resolution (logMAR) units for statistical analysis. Additionally, a Pearson correlation analysis was conducted looking at predictive factors of higher Kmax change from baseline to last visit. A 2-tailed probability of 0.05 or less was considered statistically significant. Statistical analysis was performed using SPSS for Windows version 20.0 (SPSS, Inc, Chicago, IL, USA) and Microsoft Excel 2011 (Microsoft Office, Washington, DC, USA).

## 3. Results

This study included 302 eyes (241 patients) from 1 January 2012, to 31 March 2019: 231 A-CXL and 71 I-CXL. The overall gender ratio was 2:1 (156 males/85 females). The mean follow-up was 27.2 ± 13.2 months (range 12.0 to 85.7 months). Preoperatively, the mean maximum K value (Kmax) was 51.8 ± 4.0 D with 51.6 ± 4.1 D in A-CXL versus 52.4 ± 3.4 D in I-CXL, *p* = 0.15. Preoperative pachymetry (MCT) was significantly lower in I-CXL: 395.1 ± 40.9 µm versus 436.3 ± 44.4 µm for I-CXL and A-CXL, respectively, *p* < 0.001. Table 1 reports all preoperative data within each group.

### 3.1. Changes from Baseline to Last Visit

**Figure 1:**
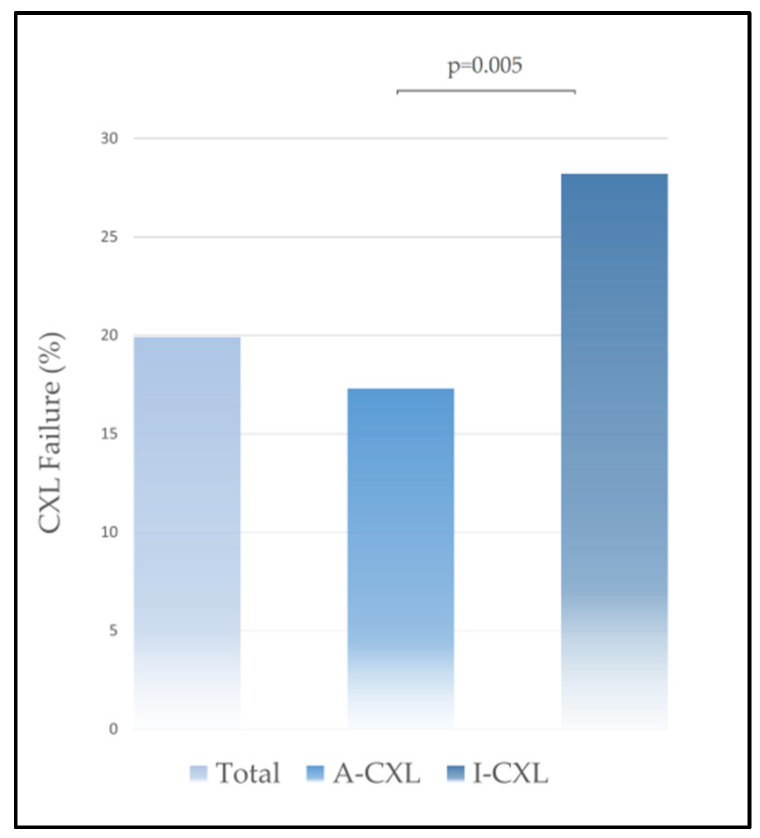
Crosslinking failure in A-CXL and I-CXL groups.

Table 2 shows the mean ± standard deviation for outcomes variation from baseline to the last visit for each group. In the overall analysis, refraction and keratometry outcomes remained stable during the follow-up. The mean change in Kmax was 0.05 ± 2.99 D, CI_95%_= [−0.17–1.20 D]: −0.19 ± 2.62 D in A-CXL versus 0.47 ± 3.06 D in I-CXL, *p* = 0.01. Kmax value remained stable throughout follow-up in the total population and in A-CXL and I-CXL groups. At the last visit, progression of Kmax equal or above 1 diopter was reported in 60 eyes (19.9%): 40 (17.3%) in group A-CXL and 20 (28.2%) in group I-CXL, *p* = 0.005 (Figure 1). The likelihood of progression after CXL was significantly higher following I-CXL versus A-CXL: RR = 1.62, CI95 = [1.02 to 2.59], *p* = 0.04. Table 3 shows the proportion of eyes that had steepening or flattening of Kmax above one diopter at the last visit for the whole cohort and for A-CXL and I-CXL groups.

### 3.2. AS-OCT Outcomes

The presence of a demarcation line (DL) was reported at 1 month postoperatively and at the last visit and was more frequent in A-CXL: 56.3% versus 31.0% for A-CXL versus I-CXL, respectively, *p* < 0.001. The mean depth of the DL at 1 month was 182.6 ± 41.1 µm with 179.1 ± 37.8 µm in the A-CXL group versus 203.0 ± 53.3 µm in the I-CXL group, *p* = 0.01. The DL depth was not correlated with the higher efficacy of the CXL procedure (Spearman r-coefficient: −0.06; *p* = 0.45).

### 3.3. Predictive Factors of Failure

Higher Kmax (r^2^ = 0.02, *p* = 0.77), Kmin (r^2^ = 0.05, *p* = 0.46), and Km values (r^2^ = 0.05, *p* = 0.41) as well as a lower age (r^2^ = 0.02, *p* = 0.77) and initial pachymetry (r^2^ = −0.08, *p* = 0.28) and visual acuity (r^2^ = 0.02, *p* = 0.74) were not associated with higher Kmax change from baseline to last visit. The main predictors of CXL failure were the I-CXL protocol (*p* = 0.005) and the absence of a DL at 1 month (*p* = 0.03), as the DL was reported in 132 eyes in the CXL success subgroup versus 23 eyes in the CXL failure subgroup.

### 3.4. Safety and Adverse Events

We report one (1.4%) case of recurrent corneal erosion syndrome following I-CXL and three (1.1%) cases of corneal abscesses following A-CXL procedure (caused by Streptococcus bovis and Staphylococcus aureus). The mean BCVA difference from baseline to the last visit was 0.01 ± 0.17 logMAR with no difference between groups (*p* = 0.92). Two eyes required deep lamellar anterior keratoplasty after A-CXL and I-CXL procedure for aggressive KC (delay before DALK= 1.5 and 1 years, age = 16.4 and 21.1 years, respectively). In the overall analysis, no significant difference in ECD was reported from baseline to last visit (3.3 ± 263.5 mm/cm^2^) and within groups. In a subgroup analysis of 51 eyes with pachymetry below 400 µm (mean = 380.3 ± 16.5 µm; range = 342–399 µm), including 30 and 21 eyes in the A-CXL and I-CXL groups, respectively, no significant difference in ECD was reported from baseline to last visit nor within groups.

## 4. Discussion

We reported a large retrospective cohort of keratoconic eyes treated by corneal CXL in a tertiary center. Although the A-CXL and I-CXL groups yielded comparable initial Kmax values, initial pachymetry was higher in the A-CXL group. This can be explained as our cohort was retrospective and is consistent with surgeons’ preferences for epi-on techniques in thin corneas cases. The success rate of A-CXL was 85.2%, and the success rate of I-CXL was 71.8%. Hence, the failure rate of the I-CXL protocol was almost two times higher than A-CXL in our cohort. The main predictive factors of failure were the I-CXL protocol and the absence of a demarcation line at 1 month.

Although numerous studies are reported in the literature concerning different A-CXL or I-CXL protocols, anterior studies yield heterogeneous results with failure rates that fluctuate between 8% and 33% [9], depending on the CXL protocol, the study design, the initial severity of the disease and the follow-up period. In 2020, Hatch and colleagues [10] showed in a prospective non-randomized study on 612 eyes that the A-CXL protocol was effective in slowing keratoconus in adults and reported a similar failure rate of 17.9% at 1 year. Similarly, Singal and colleagues [11] reported a failure rate of 21% for 204 eyes at 1 year using the same protocol. On the other hand, Mazzotta et al. reported a failure rate of 8% after A-CXL [8]. We reported a higher failure rate for 71 eyes treated with I-CXL than previously reported in smaller cohorts in the literature. In a previous study [5], we reported a failure rate of I-CXL of 20% on 40 eyes at 2 years, but initial Kmax was higher and corneal thickness was lower in this cohort as I-CXL was preferentially used for thin corneas cases to retain the epithelium intact based on our previous findings.

### 4.1. CXL for Thin Corneas

Most clinicians generally avoid CXL treatment in thin corneas with pachymetry below 400 µm after epithelial removal due to the risk of endothelial toxicity [12]. This latter point explains why I-CXL was more frequently used than A-CXL in thin corneas in our cohort. It is more advisable to use the I-CXL protocol in thin corneas with a preoperative pachymetry between 350 and 400 µm, as avoiding epithelial removal would reduce the potential endothelial damage. This is consistent with a recent survey of CXL practice patterns of members of the UK Cross-linking Consortium [13]. No endothelial damage was observed following 30 A-CXL and 21 I-CXL procedures performed on thin corneas with a total pachymetry below 400 µm (380.3 ± 16.5 µm; range = 342–399 µm). These interesting findings show that the CXL procedure in thin corneas seems effective and safe with central corneal thicknesses between 350 and 400 µm and are concordant with other previous studies conducted on thin corneas [14,15].

### 4.2. Predictive Factors for CXL Failure

Previous studies reported several preoperative predictors of keratoconus progression after CXL, such as Kmax [10], age [16,17], sex [17], cone location [18], and corneal thickness [16] affect outcomes, while other studies have not corroborated these findings [19,20]. In our cohort, we did not find a linear correlation between most of these factors and Kmax progression after CXL. Other studies on more than 100 eyes reported similar results concerning our findings on predictors of CXL failure (Greenstein and colleagues [19], Koc and colleagues [20]). The presence of a DL at 1 month was positively correlated with higher efficacy of CXL, but a lower DL depth was not a predictor of CXL failure. We described similar findings concerning the depth of the DL at 1 month after I-CXL and A-CXL in a previous study we reported [5]. In 2020, a large prospective study reported by Hatch and colleagues [10] showed that baseline visual acuity, preoperative Kmax, refraction, and central corneal thickness were all important predictors of CXL failure. These discrepancies can be explained as initial characteristics in our cohort are different from previous studies that reported these factors, with a lower mean preoperative Kmax value in our study than in the cohort reported by Hatch and colleagues [10].

### 4.3. Demarcation Line Outcomes

The presence of a DL was a predictor of CXL efficacy as it was more frequent in A-CXL (56.3%) versus I-CXL (31.0%) and less frequent in eyes with CXL failure (38.3% versus 54.0%). The DL was deeper after I-CXL (203.0 ± 53.3 µm) versus A-CXL (179.1 ± 37.8 µm). The presence of a DL in one-third of I-CXL cases reported in our study is similar to other similar studies about I-CXL protocol [6,21]. In a previous study [5], we reported the presence of a DL in only 35% of cases and a mean depth of 216.6 ± 49 mm. The absence of a DL in most of the I-CXL cases may be explained by the fact that not enough chemical reactions occur during the procedure, highlighting the necessity to enhance the I-CXL technique. Because transepithelial CXL protocols are the key to higher safety, further developments are necessary to improve I-CXL efficacy. Liao and colleagues [22] reported a failure rate at 1 year of 27.8% and 16.7% on 42 eyes using an irradiation time of 5 and 10 min, respectively. Future ongoing developments have been recently suggested by some authors and could even be combined together in order to enhance I-CXL efficacy: an increase in the current and/or duration of iontophoresis [6,23], the pulsed-light enhanced-fluence I-CXL protocol [24,25], or the oxygen-enriched I-CXL [26]. Thus, Mazzotta et al. demonstrated [24,25] that the I-CXL failure rate is explained by the fact that riboflavin concentration inside the stroma is halved after I-CXL loading compared with passive diffusion after epithelium removal. Additionally, the fluence used in I-CXL is the same used in Epi-Off without taking into account the presence of epithelium that absorbs about 30% of energy. The 40% oxygen consumption by the epithelium is left on site. Taking these parameters into account, the enhanced-fluence pulsed-light iontophoresis using 7 Joule/cm^2^ of fluence showed better results, optimizing the overall kinetic of CXL reactions [24,25].

The main limitation of this study is its retrospective design. However, this study was designed to collect real-world, long-term data to explore the long-term effectiveness and safety of these two CXL protocols with a large number of patients. Finally, both A-CXL and I-CXL protocols showed good efficacy in slowing the progression of KC with a high safety level. However, A-CXL seemed to be more effective than I-CXL in stabilizing keratoconus progression. The absence of a demarcation line on anterior segment OCT imaging at 1 month was a predictor of CXL failure. A lower threshold for minimal corneal thickness boundaries could be considered as CXL has been used in thin corneas with a high safety level. Further developments will certainly enhance iontophoresis CXL efficacy.

## Figures and Tables

**Table 1:** Preoperative data for the whole cohort and within each group.

	Total		A-CXL	I-CXL	
	Range (Min-Max)	Mean ± SD			*p*-Value
Age	12.2–59.6	25.2 ± 7.5	24.8 ± 6.9	26.7 ± 9.2	0.06
Follow-up (months)	12.0–85.7	27.2 ± 13.2	26.9 ± 13	27.9 ± 14.1	0.61
BCVA (logMAR)	−0.5–1.1	0.22 ± 0.21	0.20 ± 0.19	0.29 ± 0.23	0.50
MRSE (diopter)	−19.6–3.1	−2.5 ± 3.2	−2.5 ± 3.4	−2.6 ± 2.7	0.77
Sphere (diopter)	−15.3–6.5	−0.4 ± 3.1	−0.4 ± 3.3	−0.6 ± 2.6	0.73
Cylinder (diopter)	−9.0–4.3	−4.1 ± 2.3	−4.2 ± 2.3	−4.1 ± 2.1	0.90
Kmax (diopter)	44.6–65.8	51.8 ± 4.0	51.6 ± 4.1	52.4 ± 3.4	0.15
Kmin (diopter)	33.9–59.4	46.6 ± 3.4	46.3 ± 3.5	47.4 ± 3.1	0.16
Km (diopter)	39.3–62.4	48.7 ± 3.3	48.5 ± 3.3	49.5 ± 3	0.08
MCT (µm)	342–549	427.9 ± 46.7	436.3 ± 44.4	395.1 ± 40.9	<0.001
IOP (mmHg)	6.0–24.0	13.7 ± 3.8	13.7 ± 4	13.8 ± 3.2	0.86

SD—standard deviation; BCVA—best-corrected visual acuity; MRSE—manifest refraction spherical equivalent; Kmax—maximum keratometry; Kmin—minimum keratometry; Km—mean keratometry; MCT—minimum corneal thickness; IOP—intraocular pressure.

**Table 2:** Outcomes change from baseline to last visit for the whole cohort and within groups.

	Total			A-CXL			I-CXL			*p*-Value Intergroup	
	Initial	Final	*p*-Value	Initial	Final	*p*-Value	Initial	Final	*p*-Value		
	Mean ± SD			Mean ± SD			Mean ± SD			Initial	Final
BCVA (logMAR)	0.22 ± 0.21	0.21 ± 0.22	0.27	0.20 ± 0.19	0.19 ± 0.21	0.34	0.29 ± 0.23	0.27 ± 0.23	0.59	0.50	0.92
MRSE (diopter)	−2.5 ± 3.2	−2.4 ± 3.3	0.79	−2.5 ± 3.4	−2.3 ± 3.2	0.42	−2.6 ± 2.7	−2.7 ± 3.6	0.56	0.77	0.48
Sphere (diopter)	−0.4 ± 3.1	−0.4 ± 3.2	0.73	−0.4 ± 3.3	−0.3 ± 3.1	0.76	−0.6 ± 2.6	−0.8 ± 3.7	0.39	0.73	0.32
Cylinder (diopter)	−4.1 ± 2.3	−3.9 ± 2.0	0.10	−4.2 ± 2.3	−4.0 ± 2.0	0.21	−4.1 ± 2.2	−3.8 ± 1.8	0.26	0.90	0.38
Kmax (diopter)	51.8 ± 4.0	51.8 ± 4.8	0.82	51.6 ± 4.1	51.4 ± 4.6	0.29	52.2 ± 3.4	52.6 ± 3.8	0.06	0.15	0.01
Kmin (diopter)	46.6 ± 3.4	46.6 ± 4.1	0.86	46.3 ± 3.5	46.3 ± 3.9	0.61	47.4 ± 3.1	48.0 ± 4.8	0.36	0.16	0.005
Km (diopter)	48.7 ± 3.3	48.8 ± 4.1	0.46	48.5 ± 3.3	48.5 ± 3.9	0.89	49.5 ± 3.0	50.3 ± 4.6	0.14	0.08	0.003
MCT (µm)	427.9 ± 46.7	425.7 ± 42	0.41	436.3 ± 44.4	436.1 ± 36.4	0.94	395.1 ± 40.9	386.3 ± 38.6	0.07	<0.001	<0.001
ECD (cells/mm²)	2846.1 ± 341.7	2842.7 ± 373.7	0.83	2866.9 ± 347.6	2864.2 ± 328.2	0.87	2746.2 ± 454.3	2773.8 ± 385.8	0.54	0.22	0.10

SD—standard deviation; BCVA—best-corrected visual acuity; MRSE—manifest refraction spherical equivalent; Kmax—maximum keratometry; Kmin—minimum keratometry; Km—mean keratometry; MCT—minimum corneal thickness; ECD—endothelial cells density.

**Table 3:** Proportion of eyes with steepening or flattening of Kmax above 1 diopter at last visit for the entire cohort and for A-CXL and I-CXL groups showing a higher proportion of eyes with Kmax steepening above 3 diopters following I-CXL.

	Change Kmax	Total Eyes (%)	ACXL Eyes (%)	ICXL Eyes (%)	*p*
Kmax flattening	≥1D	64 (21)	57 (21)	7 (10)	0.02
	≥2D	28 (9)	23 (8)	5 (7)	0.5
	≥3D	16 (5)	13 (5)	3 (4)	0.9
Kmax steepening	≥1D	60 (20)	40 (15)	20 (29)	0.04
	≥2D	27 (9)	17 (6)	10 (14)	0.12
	≥3D	17 (6)	9 (3)	8 (11)	0.04

## Data Availability

Data available upon reasonable request.
